# Analysis and classification of privacy-sensitive content in social media posts

**DOI:** 10.1140/epjds/s13688-022-00324-y

**Published:** 2022-03-03

**Authors:** Livio Bioglio, Ruggero G. Pensa

**Affiliations:** University of Turin, C.So Svizzera, 185, I-10149 Turin, Italy

**Keywords:** Privacy, Text classification, Content analysis

## Abstract

User-generated contents often contain private information, even when they are shared publicly on social media and on the web in general. Although many filtering and natural language approaches for automatically detecting obscenities or hate speech have been proposed, determining whether a shared post contains sensitive information is still an open issue. The problem has been addressed by assuming, for instance, that sensitive contents are published anonymously, on anonymous social media platforms or with more restrictive privacy settings, but these assumptions are far from being realistic, since the authors of posts often underestimate or overlook their actual exposure to privacy risks. Hence, in this paper, we address the problem of content sensitivity analysis directly, by presenting and characterizing a new annotated corpus with around ten thousand posts, each one annotated as sensitive or non-sensitive by a pool of experts. We characterize our data with respect to the closely-related problem of self-disclosure, pointing out the main differences between the two tasks. We also present the results of several deep neural network models that outperform previous naive attempts of classifying social media posts according to their sensitivity, and show that state-of-the-art approaches based on anonymity and lexical analysis do not work in realistic application scenarios.

## Introduction

The Web is pervaded with user-generated contents as Internet users have multiple and increasing ways to express themselves. They can post reviews of products, businesses, services and experiences; they can share their thoughts, pictures and videos through different social media platforms; they reply to surveys, forums and newsgroups and some of them have their own blogs and web pages. Many companies are encouraging this behavior, because user-generated content has more attractive power on other users than professional contents, and this increases their engagement on web platforms. However, texts, photos and videos posted by users may harm their own and other’s privacy, thus exposing themselves (and other users) to many risks, from discrimination or cyberbullying to frauds and identity theft. Although user-generated content is often subject to moderation, also adopting automated recognition techniques such as inappropriate content [1], hate speech [2] and cyberbullying [3] detection, there is no control on the sensitivity of posted contents. It is worth noting that social media and forums are not the only platforms that store and publish private contents. Surveys, or contact/helpdesk forms are other examples where the users are free to enter any type of text and other contents, together with other more structured personal information. Often, such data need to be transferred to third parties to be analyzed, and the lack of control on free-text fields could put the privacy of respondents at risk. A common quick solution consists in totally removing all such fields or sanitizing them automatically or at hand. However, existing automatic sanitization approaches [4–6] try to replace sensitive terms belonging to specific domains (e.g., medical or criminal records) with more general ones, and rely on existing knowledge bases and natural language processing techniques such as named entity recognition and linking. In some cases, sanitization techniques destroy the informativeness (and sometimes the meaning itself) of the text.

Self-disclosure, i.e., the act of revealing personal information to others [7], is a social phenomenon that has also been extensively studied in relation with online forums [8], online support groups [9] and social media [10]. Although self-disclosure is also closely related to content sensitivity, it has often been investigated in the context of intrinsically sensitive topics, such as in forums related to health issues, intimate relationships, sex life, or forum sections explicitly devoted to people searching for support from strangers [11]. In these settings, the identity of the users is often masked by pseudonyms or entirely anonymous. Instead, general purpose social media platforms usually encourage the usage of the real identity, although this does not prevent their users from disclosing very private information [12–14]. Moreover, the sensitivity of social media texts is harder to detect, because the context of a post play a fundamental role as well. Finally, social media posts are sometimes very short; yet, they may disclose a lot of private information.

**Figure 1:**
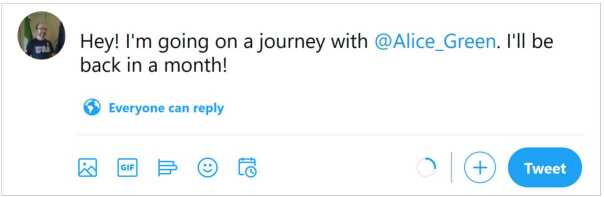
A potentially sensitive post. The post does not mention any sensitive term or topic, but discloses information about the author and his friend Alice Green, and contains hidden spatiotemporal references that are immediately clear from the context

**Figure 2:**
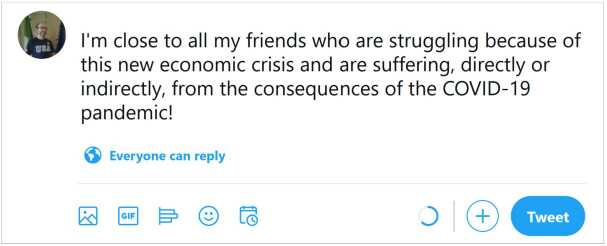
A non-sensitive post mentioning sensitive topics and terms.The post contains several sensitive terms (struggling, suffering, COVID-19) and topics (health, economic crisis), but no private information is disclosed about any specific person

To better understand the problem, let us observe the post in Fig. 1: it does not mention any sensitive term or topic, but discloses information about the author and his friend Alice Green, and contains hidden spatiotemporal references that are immediately clear from the context (the author is about to leave for a journey, which implies that he will be far from home for a month, disclosing a potentially sensitive information). On the other hand, there may exist posts that contain very sensitive terms, but are not sensitive at all, when contextualized correctly. An example is given by the post in Fig. 2, where several sensitive terms (struggling, suffering, COVID-19) and topics (health, economic crisis) are mentioned, but no private information is disclosed about any specific person. In these cases, the automatic assessment of text sensitivity could save a lot of rich information and help automate the sanitization process. Furthermore, an automatic warning system able to detect the true potential sensitiveness of a post, may help a user decide whether to share it or not.

Indeed, the problem of assessing and characterizing the sensitivity of content posted in general purpose social media has been already studied, but, due to the unavailability of specifically annotated text corpora, it has been tackled through the lens of anonymity, by assuming that sensitive contents are posted anonymously [15, 16], on anonymous platforms [17], or with more restrictive privacy settings [18], while non sensitive ones are posted by identifiable users and/or made available to everyone. However, as we pointed out in [19], anonymity and sensitivity are not straightforwardly related to each other. The decision of posting anonymously could be determined uniquely by the sensitivity of the topic, but not by the sensitivity of the posted content itself. Analogously, many non anonymous social media posts contain very private information, just because their sensitivity [12] or their visibility [14] are underestimated by the content authors. These considerations make what we call the “anonymity assumption” too simplistic, or even unrealistic in practice. Other existing annotated corpora concern posts extracted from Reddit [11] and support groups for cancer patients [8, 9]. Unfortunately, these corpora focus on very specific (and intrinsically sensitive) topics or give a very restrictive interpretation of self-disclosure: in [11], for instance, only posts disclosing personal information or feelings about the authors are annotated as sensitive. Moreover, it has a strong focus on mutually supportive communities and intimate relationships. To cope with this problems, very recently, we have introduced a more general task called *content sensitivity analysis* as a machine learning task aimed at assigning a sensitivity score to content [19]. However, in that preliminary work, we model the problem as a simple bag-of-words classification task on a very small text dataset (less than 700 social media posts) with mild accuracy results (just above the majority classifier).

In this paper, we address all the limitations of previous works by analyzing a new large corpus of nearly 10,000 text posts, all annotated as sensitive or non sensitive by humans, without assuming any implicit and forced link between anonymity and privacy. We provide an in-depth analysis of sensitive and non sensitive posts, and introduce several sequential deep neural network models that outperform bag-of-words classifiers. We also show that models trained according to the anonymity assumption do not work properly in realistic scenarios. Moreover, we also study how the problem of self-disclosure is related to ours and show that existing text corpora are not adequate to analyze the sensitivity of posts shared in general purpose social media platforms. At the best of our knowledge, this is the first work addressing the problem of directly and efficiently evaluating the real sensitivity of short text posts. It has then the potential to represent a new gold standard in content sensitivity analysis and self-disclosure, and could open new research opportunities for improving the users’ awareness on privacy and performing privacy risk assessment analysis or sanitization on data containing free text fields.

Our paper is organized as follows. In Sect. 2, we review some closely related work and discuss their limitations. We define formally our concept of privacy-sensitive content, describe how we have constructed our annotated corpus, and present the datasets used in our analysis in Sect. 3. Section 4 contains an in-depth analysis of the lexical features characterizing sensitive content in the different datasets, while, in Sect. 5, we report the results on multiple classification tasks conducted under different settings. In Sect. 6 we discuss more in detail the results of the experiments and draw some generalized conclusions. Finally, Sect. 7 concludes by also presenting some future research perspectives.

## Related work

With the success of online social networks and content sharing platforms, understanding and measuring the exposure of user privacy in the Web has become crucial [20, 21]. Thus, many different metrics and methods have been proposed with the goal of assessing the risk of privacy leakage in posting activities [22, 23]. Most research efforts, however, focus on measuring the overall exposure of users according to their privacy settings [24, 25] or position within the network [14]. Instead, the problem of characterizing and detecting the sensitivity of user-generated content, has been subject of very few studies in the last decade. One of the first work in this direction has tried to address this problem using a lexicographic approach [26, 27]. Similarly to sentiment analysis or emotion detection, in fact, linguistic resources may help identify sensitive content in texts. In their work, Vasalou *et al.* leverage prototype theory and traditional theoretical approaches to construct and evaluate a dictionary intended for content analysis. Using an existing content analysis tool applied on several text corpora, they evaluate dictionary terms according to privacy-related categories. Interestingly, the same authors note that there is no consistent and uniform theory of privacy-sensitivity.

To bypass this problem, several authors adopt a simplification: they assume that the sensitivity of contents is strictly related to the choice of posting them anonymously. This also makes the construction of annotated corpora easier, because one just needs to consider contents posted anonymously as sensitive, while posts shared with identifiable information can be considered as non sensitive. Hence, for instance, Peddinti *et al.* adopt this strategy for analyzing anonymous and non anonymous posts in a famous question-and-answer website [15]. They analyze different basic machine learning models to predict whether a particular answer will be written anonymously. Similarly, Correa *et al.* define sensitivity of a social media post as the extent to which users think the post should be anonymous [17]. They compare content posted on anonymous and non-anonymous social media sites both in terms of topics and from the linguistic point of view, and conclude that sensitivity is often subjective and it may be perceived differently according to several aspects. Very recently, the same authors have published a sanitized version of nearly 90 million posts downloaded from Whisper, an anonymous social media platforms [28]. Biega *et al.* conduct a similar study, but restrict the analysis to sensitive topics with the aim of measuring the privacy risks of the users [29]. It is worth noticing that all these studies conclude that sensitivity is subjective.

Content sensitivity has been associated to privacy settings as well: similarly to anonymity, contents posted with restricted visibility are deemed sensitive. Yu *et al.* analyze sensitive pictures by learning the object-privacy correlation according to privacy settings to identify categories of privacy-sensitive objects using a deep multi-task learning architecture [18]. They also use their model to customize privacy settings automatically and to sanitize images by blurring sensitive objects.

Text sanitization is another close research field whose goal is to find and hide personally identifiable information and simultaneously preserve text utility. To this purpose, Jiang *et al.* present an information theoretic approach to hide sensitive terms by more general but semantically related terms to protect sensitive information [30]. Similarly, Sanchez *et al.* propose several information theoretic approaches that detect and hide sensitive textual information while preserving its meaning by exploiting knowledge bases [4, 31, 32]. Iwendi *et al.*, instead, focus on unstructured medical datasets and propose a framework to completely anonymize the textual clinical records exploiting regular expressions, dictionaries and named entity recognition. Their methods is aimed at sanitizing the detected protected health information with its available generalization, according to a well-known medical ontology [5]. Finally, Hassan *et al.* use word embeddings to evaluate the disclosure caused by the textual terms on the entity to be protected according to the similarity between their vector representations [6]. All the above mentioned methods rely on the identification of named entities or quasi-identifying terms, and try to replace them with semantically close, although more general, terms. Hence, they all leverage some kind of knowledge bases or ontologies, and work well on some specific domains (e.g., on medical documents, criminal records and so on). Instead, we address a more general notion of sensitivity, that also includes texts that may reveal sensitive or simply private user’s habits, feelings or characteristics.

A closely related concept is the so-called self-disclosure, defined as the act of revealing personal information to others [7]. Self-disclosure has been widely studied well before the advent of modern social media, in particular for its implications in online support groups, online discussion boards and forums. For instance, Barak *et al.* study, among the others, the reciprocity of self-disclosure in online support groups and discussion forums showing that there are substantial differences in how people behave in these two different media types [8]. Yang *et al.*, instead, analyze the differences in the degree of positive and negative self-disclosure in public and private channels of online cancer support groups [9]. They show that people tend to self-disclose more in public channels than in private ones. Moreover, negative self-disclosure is also present more in public online support channels than in private chats or emails. To achieve these results, the authors study lexical, linguistic, topic-related and word-vector features of a relatively small annotated corpus using support vector machines. Ma *et al.* conduct a questionnaire-based mixed-factorial survey experiment to answer several questions concerning the relationships that regulate anonymity, intimacy and self-disclosure in social media [10]. They show, for instance, that intimacy always regulates self-disclosure, while anonymity tends to increase the level of self-disclosure and decrease its regulation, in particular for content of negative valence. Differently from the previous works, Jaidka *et al.* directly address the problem of self-disclosure detection in texts posted in online forums, by reporting the results of a challenge concerning a relatively large annotated corpus made up of top posts collected from Reddit [11]. Contrarily to [28], in this corpus, all posts are directly annotated according to their degree of informational and emotional self-disclosure. The authors also intend to investigate the emotional and informational supportiveness of posts and to model the interplay between these two variables. Unfortunately, this corpus is not entirely adapted to our purpose (i.e., detecting the sensitivity of text content in general purpose social media platforms) mainly for four different reasons: first, the focus is on *self*-disclosure, although a post may reveal sensitive information about other people as well; second, posts on Reddit are published using pseudonyms, while general purpose social media foster the usage of real identities; third, a large part of the posts has been extracted from a subreddit explicitly devoted to people searching for other users’ support; last but not least, all posts concern intimate relationships by design.

In conclusion, in our work, we do not make any “anonymity” or “privacy settings” assumption, since it has been shown that users tend to underestimate or simply overlook their privacy risk [12–14]. Consequently, we analyze and characterize sensitive posts directly. In a very preliminary version of our work, we tried to give a more generic definition of sensitivity [19]. However, our model was trained on very few posts and by using simple bag-of-words classifiers, thus achieving mild accuracy results. In this work, we construct a much larger and more reliable dataset of social media posts, directly annotated according to their sensitivity, and use more sophisticated and accurate models to help decide whether a post is sensitive or not. Additionally, we provide further lexical and semantic insights about sensitive and non sensitive texts.

## An annotated corpus for content sensitivity

In this section, we introduce the data that we use in our study. We first provide a conceptualization of “content sensitivity” also in relation with existing similar concepts; then, we describe how we construct our annotated corpus and provide some characterization of it.

### Privacy-sensitive content

Content sensitivity is strictly related to the concept of self-disclosure [7], a communication process by which one person reveals any kind of personal information about themself (e.g., feelings, goals, fears, likes, dislikes) to another. It has been described within the social penetration theory as one of the main factors enabling relationship development [33, 34]. Due to the peculiarities of online communication (and its differences w.r.t. face to face communication), the social and psychological implications of self-disclosure in the Internet have been extensively studied as well [35]. For its implications on user privacy, self-disclosure has also been investigated in relation with privacy awareness, policies and control [36], and some rule-based detection techniques for self-disclosure in forums have been proposed [37], leading to some relatively large annotated corpora [11].

In this paper, we refer to *content sensitivity* as a more general concept than *self-disclosure*. In [19] we gave a preliminary, subjective and user-centric definition of privacy sensitive content. In that work, we stated that a generic user-generated content object is privacy-sensitive if it makes the majority of users feel uncomfortable in writing or reading it because it may reveal some aspects of their own or others’ private life to unintended people. This definition is motivated by the fact that each social media platform has its own peculiarities and the amount and quality of social ties also play a fundamental role in regulating self-disclosure [10]. However, it has many drawbacks, since it relies on the subjective perception of users and on a notion of uncomfortableness that can also be driven by other external factors. This also conditioned the preliminary annotation of a corpus, leading to poor detection results. Consequently, in this paper, we adopt a more objective definition of privacy-sensitive content.

#### Definition 1

(Privacy-sensitive content)

A generic user-generated content is privacy-sensitive if it discloses, *explicitly or implicitly*, any kind of personal information about its author or *other identifiable persons*.

Differently from the concept of self-disclosure, our definition explicitly mention the disclosure of information concerning persons other than the author of the content. Furthermore, it also clearly includes contents that implicitly reveal personal information of any kind. For instance, the sentence “There’s nothing worse than recovering from COVID-19”, is a neutral sentence, apparently. However, it is very likely that the person who expresses this claim has also personally experienced the effects of SARS-CoV-2 infection.

### Datasets

Most previous attempts of sensitivity analysis on text contents assume that sensitive posts are shared anonymously, while non sensitive posts are associated to real social profiles. Other available corpora do not explicitly require that distinction, but have been collected in very specific domains (e.g., health support groups [9]) or focus on limited types of self-disclosure (e.g., intimate/family relationships [11]). Hence, we will consider a new generic dataset with explicit “sensitive/non-sensitive” annotations. To this purpose, we first need a corpus constituted of mixed sensitive and non-sensitive posts. Twitter is not the most suitable source for that, because most public tweets are of limited interest to our analysis, while tweets with restricted access can not be downloaded. Moreover, it is well known that users are significantly more likely to provide a more “honest” self-representation on Facebook [38, 39]. Consequently, Facebook posts are more adapted to our purposes, but contents posted on personal profiles can not be downloaded, while public posts and comments published in pages do not fit the bill as they are, in general, non sensitive. Furthermore, they would require a huge sanitization effort in order to make them available to the research community. Fortunately, one of the datasets described in [40], and released publicly, has all the required characteristics. It is a sample of 9917 Facebook posts (status updates) collected for research purposes in 2009-2012 within the myPersonality project [41], by means of a Facebook application that implemented several psychological tests. The application obtained the consent from its users to record their data and use it for the research purposes. All the posts have been sanitized manually by their curators: each proper name of person (except for famous one, such as “Chopin” and “Mozart”) has been replaced with a fixed string. Famous locations (such as “New York City” and ```Mexico”) have not been removed, either. Almost all posts are written in English, with an average length of 80 characters (the minimum and maximum length are, respectively 2 and 435 characters). Since the recruitment has been carried out on Facebook, the dataset suffers from the typical sample bias due to the Facebook environment (some groups of people might be under- or over- represented). However, the same problem applies to other datasets as well [9, 11, 28].

All 9917 posts have been proposed to a pool of 12 volunteers (7 males and 5 females, aged from 24 to 41 years, mainly postgraduate/Ph.D. students and researchers), so as to have exactly three annotations per each post. Hence, we have formed four groups, each consisting of three annotators; every group has been assigned from 2479 to 2485 posts. For each post, the volunteers had to say whether they think that the post was *sensitive*, *non-sensitive*, or of *unknown sensitivity*. The choices also include a fourth option, *unintelligible*, used, for instance, to tag posts written in a language other than English. For each category, the annotators were given precise guidelines and examples (see Table 1). According to our guidelines, a post is “sensitive” if the text is understandable and the annotator is certain that it contains information that violates a person’s privacy (not necessarily of the author of the post), because it contains, for instance: information about current or upcoming moves, on events in the private sphere, on health or mental status; information about one’s habits or that can help geolocalize the author of the post or other people mentioned; information on the sentimental status; considerations that may hint at the political orientation or religious belief.

**Table 1:** Guidelines and examples for the annotations

Category	Guidelines	Examples
Sensitive	A post is “sensitive” if the text is understandable, i.e., written in clear English, and the annotator is certain that it contains information that violates a person’s privacy, not necessarily of the author of the post. A text violates a person’s privacy if contains the following types of information (non-exhaustive list): • current or upcoming moves; • information on events in the private sphere; • information on health or mental status; • information about one’s habits; • information that can help geolocalize the author of the post or other people mentioned; • information on the sentimental status; • considerations that may hint at the political orientation or religious belief of a mentioned person. In general, given the subjectivity of the topic, a post can be sensitive if the person reading it feels discomfort due to the private content it contains (and not to other moral considerations).	“...heading to the gym with *PROPNAME*, *PROPNAME* and my sista!!” “is feeling uninspired and unmotivated. Can someone else please pay her bills and move her into her new apartment?” “is very sore and very tired...” “Just wanted to thank everyone for all the support (and great tips) yesterday, it meant a lot! made it through yesterday without smoking at all...and still going strong! :)” “Lazy day around the house after the family has left.” “ARGH. 2 whole years! Congratulations, *PROPNAME*! You’ve tolerated me for a total of 730 days! Plus ‘getting to know you’ time... hahaha!” “is shaking his head wondering when some of his conservative christian friends became so hate filled that they will join any anti-obama group on facebook.”
Non sensitive	A post is “non-sensitive” if the text is understandable, i.e., written in clear English, and the annotator is sure that it does not contain information that violates privacy, according to the indications of the “sensitive” category.	“Fabulous weekend :-)” “When we are no longer able to change a situation – we are challenged to change ourselves. Viktor E. Frankl” “loves summer evenings”
Unknown	A post is of “unknown sensitivity” if the text is understandable, i.e., written in clear English, but the annotator is unable to tell if it contains information that is sensitive for privacy, because (non-exhaustive motivations): • the context is not sufficient to understand the sensitivity of the message; • the post is incomplete, i.e., the text does not contain the whole post, and from the available portion one is unable to understand its sensitivity; • the post contains a reference to a media (an image, a link, a GIF) which is considered essential for understanding the message, if the text alone is not sufficient to understand its sensitivity.	“black” “Goodbye *PROPNAME*. :(“ “I know 6 sick people at the moment, and now I’m...” “Check out what I’ve got written for The Book of *PROPNAME*. [link]”
Unintelligible	A post can be marked as “unintelligible” when: • it is written with slang/abbreviations or a grammar that does not render it understandable from a lexical point of view; • the post is written in a language other than English.	“hooked on PBS” “fml” “wahhhh,. di na ko. hurot na jud ako kwarta aning AI. huhuhu” “Pas de mauvaise nouvelle pour l’instant! Je presume donc que c’est une bonne chose!”

At the end of the period allowed for the annotation, all volunteers have accomplished their assigned task and we have computed some statistics regarding their agreement. In details, for each group, we have computed the Fleiss’ *κ* statistics [42], which measure the reliability of agreement between a fixed number of annotators. The results (reported in Table 2) show fair to moderate agreement in all groups, also considering that the number of possible categories is four. This result also demonstrate that the task of deciding whether a post is sensitive or not is not straightforward, as shown by the percentage of identical annotation in each groups: overall, at least 93.91% of posts have at least two identical annotations, but the percentage drops down to 42.97% if we look for the perfect agreement (three unanimous annotators). Apparently, there are differences among the four groups, but they are smoothed by only considering posts with at least two “sensitive” or “non-sensitive” tags, as we will precise later.

**Table 2:** Agreement computed according to Fleiss’ *κ*

Group	Fleiss’ *κ*	2 agree	3 agree
Group 1	0.34	94.44%	45.00%
Group 2	0.23	93.75%	35.14%
Group 3	0.22	90.96%	35.18%
Group 4	0.42	96.49%	56.56%
Mean	0.31	93.91%	42.97%

In Table 3 we report the details of the annotations. Each column reports the number of posts that received exactly one, two or three annotations for each class. From this table it emerges how the majority (7923) of posts have been annotated at least once as non-sensitive, while the number of posts that have received at least one “sensitive” annotation are much less (5826). In addition, the number of posts with unknown sensitivity drops drastically from 1529 to 7 when the number of annotations considered increases from one to three. This means that for almost all posts (except unintelligible ones) at least one annotator was able to determine its sensitivity.

**Table 3:** Details of the annotations. The last column contains the number of posts receiving at least one annotation for each class

Class	1 annot.	2 annot.	3 annot.	Sum
Sensitive	2490	1892	1444	5826
Non-sensitive	2494	2827	2602	7923
Unknown	1529	183	7	1719
Unintelligible	357	150	208	715
Total	6870	5052	4261	–

Starting from all the annotations, we generate two datasets. The first one contains all those posts that received at least two “sensitive” or “non-sensitive” annotations and we call it *SENS2*. The second, called *SENS3* contains all those posts that received exactly three “sensitive” or “non sensitive” annotations. By operating this choice, we exclude automatically all posts that have been annotated as “unknown” or “unintelligible” by at least two annotators. Notice that the portion of sensitive posts is almost the same in both samples. The details of these two datasets are reported in Table 4. The average length of the posts (in terms of number of words) is relatively small (15 words, on average), a typical characteristic of social media text contents, but there is a high variability (some posts are more than 85 words long).

**Table 4:** Details on the datasets used

Dataset	# posts	# sens	# ns	Avg # words
SENS2	8765	3336	5429	15.11 ± 12.58
SENS3	4046	1444	2602	15.40 ± 12.67
OMC	17,860	10,793	7067	15.58 ± 11.00
WH+TW	8765	3336	5429	13.08 ± 8.26

For comparison reasons, we also use two additional datasets. The first consists of top posts extracted from two subreddits in Reddit [11]:^1^ “r/CasualConversations”, a sub-community where people are encouraged to share what’s on their mind about any topic; “r/OffmyChest”, a mutually supportive community where deeply emotional things are shared. By design, all posts mention one of the following terms: *boyfriend*, *girlfriend*, *husband*, *wife*, *gf*, *bf*. The annotators were required to annotate each post according to the amount of emotional and informational disclosure it contains. Here, we consider all posts that do not disclose anything as “non sensitive”; all remaining posts are tagged as “sensitive”, in accordance with the choices made for annotating our dataset. We consider all the 12,860 labeled training data samples and the 5000 labeled test data samples. Overall, 10,793 posts are labeled as “sensitive”, and 7067 as “non sensitive”. All the details are given in Table 4. The reader is referred to [11] for further details about this dataset.

The second dataset is an anonymity-based corpus following the example of [17], where sensitive posts are constituted of anonymous posts shared on Whisper^2^ (a popular social media platform allowing its users to post and share photo and video messages anonymously), while non-sensitive posts are taken from Twitter. Here, we generate ten samples, each consisting of a subset of 3336 sensitive posts selected randomly from a large collection of sanitized Whisper posts [28],^3^ and a subset of 5429 non-sensitive posts randomly picked from a large collection of tweets [43]. The numbers of sensitive and non-sensitive posts have been chosen to mimic the distribution observed in dataset *SENS2*. We filter out posts containing retweets or placeholders, and that are shorter than 9 characters or not written in English (according to the fastText model [44]). Then, from each remaining post, we remove any mention and hashtag, in order to obtain samples of posts similar to the ones in *SENS2* and *SENS3*. The ten samples are needed to limit any sampling bias.

## Understanding sensitivity

In this section, we analyze our data in detail with the aim of characterizing sensitive and non-sensitive posts from a linguistic point of view. The goal of this analysis is to understand whether lexical features may help distinguish sensitive and non-sensitive content.

### Analysis of the words

As first analysis, we extract the most relevant terms from each class of posts in all datasets considered in our study. To this purpose, all terms are first stemmed. Then, we compute the total number of their occurrences and their relative frequency for all classes as the number of occurrences of each word in each class (sensitive and non-sensitive) divided by its total number of occurrences. To avoid any bias, the number of occurrences and the relative frequency are computed on 10 random samples consisting of 500 sensitive and 500 non-sensitive posts. The results are then averaged on the 10 samples. Only words occurring at least 30 times are considered. The top-20 words ranked according to their average relative frequency in each class are shown in Tables 5, 6, 7 and 8. It is worth noting that, for the sensitive class, relative percentages are in general much higher for *WH*+*TW* than *SENS2*, *SENS3* and *OMC*. Moreover, emergent words in *WH*+*TW* are mostly related to personal relationships, while most emergent terms in *SENS2* and *SENS3* are more generic and related to everyday life. This highlights one of the limitations of previous work based on anonymity, such as [17], i.e., the fact that using different sources to gather anonymous and non-anonymous posts introduces a bias also in terms of discussion topics. Moreover, Table 7 shows the intrinsic bias of dataset *OMC*: the most prominent words for the sensitive class are related to friendship and personal feelings and wishes (e.g., *friend*, *feel*, *would*).

**Table 5:** Most relevant words for each class in dataset **SENS2**

Sensitive				Non-sensitive			
Overall rank	Word	Overall count	Relative frequency	Overall rank	Word	Overall count	Relative frequency
22	home	33.40 ± 5.74	88.05 ± 5.62	8	love	45.00 ± 7.77	59.86 ± 5.92
26	tomorrow	31.40 ± 4.58	80.36 ± 6.81	10	one	44.50 ± 7.46	56.72 ± 8.67
29	tonight	30.20 ± 5.49	75.78 ± 5.95	19	need	33.70 ± 3.71	56.52 ± 4.91
27	week	30.90 ± 3.00	75.17 ± 5.40	6	like	55.50 ± 7.79	55.10 ± 5.52
9	back	44.70 ± 7.01	74.95 ± 7.04	13	new	40.50 ± 5.32	52.94 ± 6.83
5	work	56.80 ± 6.03	74.46 ± 4.95	20	make	33.70 ± 7.45	52.60 ± 8.10
15	night	37.30 ± 6.86	71.56 ± 7.04	16	think	36.30 ± 5.87	52.57 ± 8.28
1	go	97.40 ± 9.75	67.61 ± 5.45	25	cant	31.60 ± 6.33	48.57 ± 6.53
12	today	42.30 ± 4.79	66.66 ± 5.14	7	time	51.40 ± 8.41	46.59 ± 7.41
0	propnam	123.10 ± 11.53	65.36 ± 4.62	14	good	40.00 ± 7.82	46.02 ± 7.43
4	im	58.20 ± 6.53	62.80 ± 6.88	28	happi	30.30 ± 5.68	45.41 ± 10.64
3	day	72.80 ± 10.27	62.52 ± 6.28	11	want	44.40 ± 4.53	45.02 ± 9.49
17	feel	34.00 ± 6.65	62.38 ± 5.86	24	come	32.40 ± 5.44	42.50 ± 6.18
18	see	33.90 ± 6.30	60.74 ± 5.49	23	know	33.40 ± 7.44	41.55 ± 10.81
2	get	80.50 ± 6.60	58.63 ± 2.28	21	got	33.60 ± 8.10	41.46 ± 8.29
21	got	33.60 ± 8.10	58.54 ± 8.29	2	get	80.50 ± 6.60	41.37 ± 2.28
23	know	33.40 ± 7.44	58.45 ± 10.81	18	see	33.90 ± 6.30	39.26 ± 5.49
24	come	32.40 ± 5.44	57.50 ± 6.18	17	feel	34.00 ± 6.65	37.63 ± 5.86
11	want	44.40 ± 4.53	54.98 ± 9.49	3	day	72.80 ± 10.27	37.48 ± 6.28
28	happi	30.30 ± 5.68	54.60 ± 10.64	4	im	58.20 ± 6.53	37.20 ± 6.88

**Table 6:** Most relevant words for each class in dataset **SENS3**

Sensitive				Non-sensitive			
Overall rank	Word	Overall count	Relative frequency	Overall rank	Word	Overall count	Relative frequency
13	home	43.50 ± 4.33	92.82 ± 4.94	24	peopl	33.00 ± 3.13	70.34 ± 7.09
15	tomorrow	38.30 ± 3.83	90.45 ± 4.21	9	one	48.60 ± 6.59	66.78 ± 7.59
29	tonight	31.20 ± 6.25	86.73 ± 3.92	11	love	45.60 ± 5.04	64.11 ± 5.28
30	weekend	30.50 ± 4.79	85.68 ± 4.12	19	think	34.60 ± 3.92	63.46 ± 5.11
4	work	56.90 ± 6.59	84.55 ± 5.39	22	dont	33.50 ± 5.76	61.47 ± 5.66
5	back	56.10 ± 5.69	80.52 ± 3.67	7	like	55.60 ± 8.86	61.36 ± 6.06
1	go	110.00 ± 10.58	73.62 ± 2.63	18	make	34.60 ± 4.01	58.45 ± 8.25
0	propnam	149.10 ± 12.51	73.12 ± 5.17	23	happi	33.30 ± 3.68	57.76 ± 8.16
26	night	32.10 ± 5.47	70.74 ± 11.19	27	know	32.10 ± 6.89	55.75 ± 6.38
10	today	45.70 ± 3.80	69.28 ± 4.51	14	new	40.00 ± 6.46	49.41 ± 6.27
20	got	34.50 ± 2.42	67.46 ± 7.07	16	good	37.90 ± 7.77	47.78 ± 4.71
21	come	33.80 ± 5.63	67.43 ± 6.09	17	want	36.10 ± 6.12	46.69 ± 6.84
6	im	55.90 ± 6.59	67.15 ± 4.28	28	feel	31.40 ± 5.76	42.22 ± 10.66
3	day	72.50 ± 6.75	67.09 ± 3.33	8	time	54.60 ± 7.09	41.16 ± 6.60
12	see	44.60 ± 7.00	63.17 ± 5.80	25	cant	32.60 ± 4.17	38.58 ± 5.07
2	get	87.20 ± 8.20	62.64 ± 4.44	2	get	87.20 ± 8.20	37.36 ± 4.44
25	cant	32.60 ± 4.17	61.42 ± 5.07	12	see	44.60 ± 7.00	36.83 ± 5.80
8	time	54.60 ± 7.09	58.84 ± 6.60	3	day	72.50 ± 6.75	32.92 ± 3.33
28	feel	31.40 ± 5.76	57.78 ± 10.66	6	im	55.90 ± 6.59	32.85 ± 4.28
17	want	36.10 ± 6.12	53.31 ± 6.84	21	come	33.80 ± 5.63	32.57 ± 6.09

**Table 7:** Most relevant words for each class in dataset **OMC**

Sensitive				Non-sensitive			
Overall rank	Word	Overall count	Relative frequency	Overall rank	Word	Overall count	Relative frequency
1	im	79.70 ± 7.07	71.51 ± 3.59	2	dont	75.30 ± 8.76	54.77 ± 7.02
29	year	30.10 ± 2.77	70.39 ± 12.45	17	your	39.20 ± 5.75	54.17 ± 7.17
20	much	34.30 ± 6.52	68.82 ± 6.39	25	way	31.40 ± 5.25	53.71 ± 7.79
26	friend	31.20 ± 7.15	67.46 ± 8.38	18	good	38.20 ± 4.92	53.52 ± 9.68
14	realli	43.50 ± 6.38	63.46 ± 7.29	27	that	30.50 ± 6.26	52.89 ± 6.82
23	work	33.10 ± 5.34	61.98 ± 13.41	24	tri	31.90 ± 6.81	50.95 ± 8.36
21	even	34.20 ± 4.59	61.95 ± 10.70	8	peopl	55.60 ± 5.93	49.88 ± 5.09
16	life	42.00 ± 6.94	61.80 ± 5.75	10	think	48.50 ± 4.09	48.99 ± 9.29
7	go	56.80 ± 6.61	59.78 ± 5.72	28	person	30.20 ± 6.32	48.53 ± 11.36
15	would	42.20 ± 7.96	59.32 ± 4.06	22	need	33.60 ± 4.81	47.45 ± 8.00
5	know	60.00 ± 4.74	58.69 ± 4.51	9	thing	55.40 ± 7.31	47.25 ± 5.91
4	feel	63.20 ± 7.15	57.82 ± 5.32	12	make	46.80 ± 5.73	46.65 ± 10.65
11	want	48.00 ± 8.10	57.72 ± 8.62	13	one	45.00 ± 9.49	44.14 ± 6.84
0	like	91.70 ± 8.15	57.22 ± 4.25	6	time	57.10 ± 6.87	43.91 ± 7.14
19	love	37.50 ± 6.02	56.39 ± 7.99	3	get	74.10 ± 7.32	43.85 ± 7.06
3	get	74.10 ± 7.32	56.16 ± 7.06	19	love	37.50 ± 6.02	43.61 ± 7.99
6	time	57.10 ± 6.87	56.09 ± 7.14	0	like	91.70 ± 8.15	42.78 ± 4.25
13	one	45.00 ± 9.49	55.86 ± 6.84	11	want	48.00 ± 8.10	42.28 ± 8.62
12	make	46.80 ± 5.73	53.35 ± 10.65	4	feel	63.20 ± 7.15	42.18 ± 5.32
9	thing	55.40 ± 7.31	52.75 ± 5.91	5	know	60.00 ± 4.74	41.31 ± 4.51

**Table 8:** Most relevant words for each class in *WH*+*TW*

Sensitive				Non-sensitive			
Overall rank	Word	Overall count	Relative frequency	Overall rank	Word	Overall count	Relative frequency
295	lesbian	41.70 ± 6.36	97.49 ± 2.75	190	ni**a	58.80 ± 8.23	100.00 ± 0.00
357	bi	33.20 ± 6.63	97.43 ± 2.41	269	rt	44.60 ± 9.19	99.78 ± 0.69
91	chat	101.10 ± 7.82	96.59 ± 1.49	194	tweet	57.80 ± 7.08	99.24 ± 1.36
281	whisper	43.20 ± 7.39	95.85 ± 2.63	219	da	52.90 ± 11.61	98.94 ± 1.53
73	boyfriend	117.30 ± 15.94	95.19 ± 1.96	376	kno	30.60 ± 5.17	98.60 ± 2.53
142	male	71.00 ± 7.54	95.09 ± 3.00	169	twitter	63.50 ± 6.59	98.18 ± 1.50
182	relationship	60.70 ± 11.44	93.27 ± 2.87	349	snow	34.30 ± 5.31	97.63 ± 2.29
249	18	47.30 ± 6.38	92.92 ± 3.63	314	wat	38.70 ± 7.56	97.20 ± 2.80
218	ex	53.20 ± 6.36	92.50 ± 3.08	121	lmao	79.80 ± 6.88	97.03 ± 1.39
237	girlfriend	49.40 ± 6.20	92.17 ± 3.65	287	jus	42.40 ± 6.72	96.90 ± 2.79
62	sex	136.80 ± 13.17	91.40 ± 2.78	159	wit	66.40 ± 6.47	96.79 ± 2.14
381	attract	30.30 ± 7.53	91.30 ± 3.81	289	yea	42.20 ± 8.42	96.30 ± 3.01
113	femal	86.00 ± 8.96	90.98 ± 3.01	257	smh	46.40 ± 7.52	95.63 ± 3.38
364	older	32.00 ± 5.72	89.81 ± 4.24	174	bout	62.30 ± 6.38	94.98 ± 2.96
288	f	42.30 ± 7.09	88.64 ± 5.65	144	ya	70.90 ± 5.26	94.43 ± 3.17
157	messag	66.80 ± 8.04	87.81 ± 4.99	3	u	613.90 ± 31.07	93.99 ± 0.93
167	gay	64.40 ± 5.78	86.50 ± 5.51	66	ur	125.10 ± 17.70	93.37 ± 2.98
373	bf	30.80 ± 5.47	86.11 ± 6.56	185	yall	59.20 ± 4.32	93.30 ± 2.87
374	cheat	30.80 ± 9.10	84.84 ± 9.58	263	lil	45.60 ± 6.38	92.81 ± 3.51
327	secret	37.20 ± 7.45	84.56 ± 6.29	6	lol	520.20 ± 30.12	92.36 ± 1.08

### Analysis of the lexical features

Similarly as in [17], we categorize all words contained in each post into different dictionaries provided by LIWC [45]. LIWC is a hierarchical linguistic lexicon that classifies words into meaningful psychological categories: for each post, LIWC counts the percentage of words that belong to each psychological category. In addition, we also account for another, more specific, lexical resource, i.e., the *Privacy Dictionary* [26, 27]. It consists of dictionary categories derived using prototype theory according to traditional theoretical approaches to privacy. The categories, together with some example of words, are presented in Table 9.

**Table 9:** Categories of the Privacy Dictionary [26]

Category name	Examples of words	Ratio SENS2	Ratio SENS3	Ratio OMC	Ratio WH+TW
*NegativePrivacy*	bully*, troubled, interfere	0.55	0.43	1.06	1.67
*Restriction*	block, hidden, quiet	0.90	0.80	1.00	2.24
*NormsRequisites*	consent, respect, discrete	0.24	0.05	1.02	7.75
*OutcomeState*	freedom, separation, alone	0.81	1.05	1.48	1.45
*OpenVisible*	post, display, accessible	0.56	0.40	0.83	1.54
*PrivateSecret*	secret, intimate, data	0.43	0.53	0.95	2.24
*Intimacy*	family, friend, group	1.24	1.30	1.51	3.99
*Law*	criminal, illegal, offence	1.96	4.25	1.00	0.89

Given 10 random samples consisting of 500 sensitive and 500 non-sensitive posts, we calculate the average percentage of sensitive and non-sensitive posts that contains words belonging to each dictionary as well as the sensitive to non-sensitive ratio for each dictionary. For the psychological categories, we only list the dictionaries whose ratio exceeds 1.3 (thus, it is over-represented in sensitive posts) or is below 0.7 (i.e., it is under-represented in sensitive posts) in each dataset. The results are shown in Table 10 (categories with high sensitive to non sensitive ratio are presented in bold), while the ratios for privacy-related categories are all reported in Table 9. It is worth noting that the number of relevant dictionaries in Table 10 differs significantly from one dataset to another: it is minimum in *SENS2* and maximum in *WH*+*TW*. Interestingly, some categories are relevant in all datasets (e.g., some personal pronouns, family, friends and female), while other ones are specific to individual corpora (anxiety and feelings appear only in *OMC* and *WH*+*TW*, money only in *SENS2* and *SENS3*). Overall, lexical features seems to help discriminate better *OMC* and *WH*+*TW* datasets rather than ours, and this observation is even more evident for the Privacy Dictionary (Table 9). In our data, with the exception of categories Law and Intimacy, almost all privacy categories are less represented in sensitive posts than in non-sensitive ones (ratios are less than one). Instead, almost all privacy categories are over-represented in sensitive posts belonging to *WH*+*TW*. In *OMC*, ratios are in general closer to one. These results confirm that relying on the anonymity of sources may introduce too much lexical bias, while considering sensitivity directly show less distinguishing lexical properties.

**Table 10:** Psychological categories of LIWC [45]

Dataset	Relevant dictionaries
SENS2	we, you, **shehe**, they, **family**, **friend**, **female**, **health**, **focusfuture**, **motion**, **space**, **time**, **work**, **leisure**, **home**, **money**
SENS3	**i**, we, you, **shehe**, they, **conj**, interrog, **number**, anger, **sad**, **family**, **female**, insight, cause, certain, hear, **health**, **sexual**, **ingest**, **achieve**, **focusfuture**, **motion**, **space**, **time**, **work**, **leisure**, **home**, **money**, relig, **netspeak**, **filler**
OMC	**i**, you, **shehe**, **they**, **conj**, **negate**, **compare**, **interrog**, **number**, **quant** **negemo**, **anx**, **sad**, **social**, **family**, **friend**, **female**, **male**, **feel**, **bio**, **body**, **health**, **sexual**, **affiliation**, **focuspast**, **home**, **relig**, **death**, **informal**, **swear**
WH+TW	**i**, you, **shehe**, **conj**, **negate**, **compare**, **interrog**, **number**, **quant**, **negemo**, **anx**, **sad**, **social**, **family**, **friend**, **female**, **male**, **cogproc**, **insight**, **cause**, **discrep**, **tentat**, **certain**, **differ**, **feel**, **bio**, **body**, **health**, **sexual**, **affiliation**, **risk**, work, relig, **death**, informal, netspeak, assent, nonflu

This consideration is confirmed by a further experiment conducted to verify whether lexical features can help discriminate sensitive posts against non-sensitive ones. To this purpose, we set up a simple binary classification task, using a logistic regression (LR) classifier, a support vector machine (SVM) classifier with linear kernel, and a Random Forests (RF) classifier with default parameters. Each dataset is randomly divided into training (75%), validation (15%) and test (10%) sets: the same sets will be employed in each experiment presented in this paper. Here, the training set is used for training the model, and the test set for performance evaluation. We train and test the classifiers on different feature sets: the one including all dictionaries, the one including only psychological dictionaries, and the one consisting only of privacy categories. Each post is then represented by a vector whose values are the percentage of words in the post belonging to each dictionary. Values are standardized to have zero mean and unit variance. According to the results presented in Table 11, *WH*+*TW* seems to take greater advantage of lexical features w.r.t. all other datasets (in particular, *OMC* and the equally-sized *SENS2*). Another important observation concerns the impact of privacy categories on classification. Apparently, some classification results are penalized by these features and, when the classifier is trained on privacy categories only, the performances drop drastically to those of the majority classifier. One explanation is that such a dictionary is built upon technical documents and is not intended as a general-purpose lexical resource, although some categories also applies to our data (e.g., *Intimacy*). This is also confirmed by the fact that this feature space is very sparse (non-zeros are around 2% in all datasets). Nevertheless, in this analysis we have considered it because this is the only existing lexical resource having a specific focus on privacy.

**Table 11:** Classification results (macro averaged F1-score) using dictionary features. Results on WH+TW are averaged on ten samples

Dataset	Class.	All dict.	Psych. dict.	Priv. Dict
SENS2	LR	0.64	0.65	0.38
	RF	0.65	0.66	0.41
	SVM	0.64	0.65	0.38
SENS3	LR	0.72	0.72	0.39
	RF	0.70	0.69	0.42
	SVM	0.70	0.72	0.39
OMC	LR	0.63	0.63	0.38
	RF	0.67	0.66	0.40
	SVM	0.62	0.63	0.38
WH+TW	LR	0.78 ± 0.01	0.77 ± 0.01	0.46 ± 0.01
	RF	0.78 ± 0.01	0.78 ± 0.01	0.49 ± 0.02
	SVM	0.77 ± 0.01	0.77 ± 0.01	0.46 ± 0.01

### In-depth analysis of dictionary-based classification results

To better understand the behavior of the classifiers, we analyze in detail the performance on the different classes (the sensible and the non-sensible ones), in terms of F1-score and for each dataset, considering the best performing classifiers according to the macro-averaged F1-score (see Table 11). The results are reported in Table 12. As expected, the majority class (the non-sensible one for every dataset except *OMC*) is the one for which the classifiers are the most accurate. However, from the classification point of view, *WH*+*TW* is the easiest dataset to analyze, as the two classes are better identified than in any other dataset, while on *SENS2* and *OMC* the best classifiers achieve similar performances, slightly better than the majority voting classifier for the most frequent class. For such datasets, using dictionaries does not provide a reliable way to differentiate the two classes.

**Table 12:** Detailed classification results (F1-score) using dictionary features with the best classifier. Results on WH+TW are averaged on ten samples

Dataset	Best class.	F1(sens.)	F1(non-sens.)	F1(macro)
SENS2	RF	0.53	0.78	0.66
SENS3	LR	0.62	0.82	0.72
OMC	RF	0.75	0.56	0.66
WH+TW	RF	0.70 ± 0.01	0.84 ± 0.00	0.78 ± 0.01

Finally, we inspect the logistic regression classifier to identify the most relevant features for the sensitive class in each dataset. In Table 13 we report the top-20 relevant features together with the corresponding coefficients (the logarithms of the odds ratios). The results seem to confirm the conclusions reached with the previous experiments (feature names with capital initials are from the Privacy Dictionary [26, 27]), but as further analysis, we compute the Spearman’s rank correlation coefficient (referred to as *ρ* in the following) among the different feature coefficient vectors in order to investigate the similarities among the different models. The results of this analysis show that, not surprisingly, the two most similar logistic regression models are those computed on *SENS2* and *SENS3* ($\rho =0.757$). However, more interestingly, the model computed on *WH*+*TW* is more similar to the one computed on *OMC* ($\rho =0.25118$) than to those computed on *SENS2* and *SENS3* ($\rho =0.1165$ and $\rho =-0.0007$). This shows that the types of sensitiveness captured by *OMC* and *WH*+*TW* have something in common: this is probably due to the fact that the content of sensitive posts for both datasets is mostly related to family and intimate relationships. Finally, it is worth noting that the coefficients computed on *OMC* are more correlated with those computed on *SENS3* ($\rho =0.3277$) than with those returned for *SENS2* ($\rho =0.1461$). This can be explained by the fact that the annotators’ agreement on *SENS3* is the highest one: as a consequence, only highly sensitive posts (such as the ones tagged as sensitive in *OMC*, by construction) are marked as such. However, as already declared, we are interested in a more general concept of content sensitivity which does not rely on the most personal and intimate aspects of the human’s life only.

**Table 13:** Top-20 relevant features and their coefficients computed by the logistic regression classifier for the sensitive class

Dataset	Feture name (coefficient value)
SENS2	Law (0.1075), family (0.0968), OutcomeState (0.0725), health (0.0697), i (0.0617), informal (0.0586), Restriction (0.0537), affect (0.0486), shehe (0.0479), home (0.0463), prep (0.0450), focusfuture (0.0431), ipron (0.0421), Intimacy (0.0408), NormsRequisites (0.0356), ppron (0.0289), work (0.0265), conj (0.0257), friend (0.0228), anx (0.0212)
SENS3	Law (0.1928), family (0.1639), affect (0.1133), OutcomeState (0.1006), informal (0.0900), health (0.0865), home (0.0836), Restriction (0.0822), pronoun (0.0822), focusfuture (0.0812), prep (0.0665), i (0.0628), shehe (0.0543), conj (0.0502), money (0.0487), friend (0.0454), reward (0.0417), sad (0.0388), number (0.0303), differ (0.0283)
OMC	pronoun (0.1831), family (0.0552), OutcomeState (0.0461), i (0.0398), Intimacy (0.0286), negemo (0.0283), bio (0.0263), conj (0.0236), friend (0.0216), sexual (0.0203), feel (0.0189), relativ (0.0188), informal (0.0177), male (0.0169), prep (0.0148), number (0.0145), adj (0.0142), quant (0.0142), posemo (0.0134), female (0.0107)
WH+TW	sexual (0.1358 ± 0.0312), female (0.1033 ± 0.0103), PrivTtl (0.0978 ± 0.0401), i (0.0833 ± 0.0489), ipron (0.0806 ± 0.1230), male (0.0744 ± 0.0113), cogproc (0.0703 ± 0.0087), ppron (0.0654 ± 0.1355), feel (0.0547 ± 0.0209), social (0.0534 ± 0.0063), conj (0.0483 ± 0.0080), number (0.0446 ± 0.0046), see (0.0427 ± 0.0252), prep (0.0414 ± 0.0051), affect (0.0355 ± 0.0399), article (0.0306 ± 0.0079), body (0.0295 ± 0.0099), health (0.0256 ± 0.0135), quant (0.0242 ± 0.0059), affiliation (0.0242 ± 0.0156)

## Classifying posts according to their sensitivity

In this section, we provide the details of the experiments conducted within different classification scenarios, where the learning algorithms are applied directly on (embeddings of) text data. Our goal is to measure the possible gain of applying recent state-of-the-art text classification techniques that consider text as sequences, over the usage of features extracted from dictionaries. In particular, we compare several different convolutional and recurrent neural networks architectures, a transformer-based neural network technique and, in addition, we also consider some baselines consisting in applying standard classifiers on bag-of-words representations of the datasets, similarly as in our previous work [19].

More in detail, we apply four different classifiers for each dataset: a one-dimensional Convolutional Neural Network (CNN), a Recurrent Neural Network (RNN) with gated recurrent unit (GRU) nodes, a RNN with long short-term memory (LSTM) nodes, and BERT [46], a pre-training transformer-based network designed for learning language representation models. The CNN models have an embedding layer, followed by one or two one-dimensional convolutional layers (all with kernel size 8, Rectified Linear Unit as activation function, batch normalization and global average pooling), one or two dense layers, and one dense layer of 2 nodes with *softmax* activation function. The exact number of nodes per level of each model is reported in Table 14. The RNN models consist of one embedding layer, followed by one or two recurrent layers, one or two dense layers, and, finally, one dense layer of 2 nodes with *softmax* activation function. The number of layers and nodes of each model is reported in Table 15. The embedding layer projects each word of the input text into a word vector space: we use two different word embeddings pre-trained on Twitter data using GloVe [47].^4^ Each recurrent layer is bidirectional, and each layer has a dropout equals to 0.5. Instead, for each dataset, BERT is trained with a learning rate equal to $5 \cdot 10^{-5}$ and early stopping on the accuracy of the validation set, with patience equals to 5. Finally, the bag-of-words (BoW) models consists of standard classifiers trained on *tfidf* features extracted from text data after applying stemming and removing stopwords. We use the same classifiers as in Sect. 4.2, i.e., a logistic regression (LR) classifier, a support vector machine (SVM) classifier with linear kernel, and a random forests (RF) classifier, all trained with default parameters. For all models, we use the same training, validation and test sets described in Sect. 4.

**Table 14:** Detailed composition (number of neurons) of the Convolutional Neural Networks

Model name	Node type	Size of the emb. layer	Size of conv. layer 1	Size of conv. layer 2	Size of dense layer 1	Size of dense layer 2
CNN1	1D-CNN	100	256	–	256	–
CNN2	1D-CNN	100	128	–	128	–
CNN3	1D-CNN	100	256	128	64	32
CNN4	1D-CNN	200	128	128	128	128

**Table 15:** Detailed composition (number of neurons) of the Recurrent Neural Networks

Model name	Node type	Size of the emb. layer	Size of rec. layer 1	Size of rec. layer 2	Size of dense layer 1	Size of dense layer 2
RNN1	GRU	100	128	128	128	128
RNN2	LSTM	100	256	128	64	32
RNN3	GRU	200	128	–	128	–
RNN4	LSTM	200	128	128	128	128

In our experiments, we use Python implementations of the algorithms of Keras, scikit-learn,^5^ and ktrain [48] libraries. All experiments are executed on a server with 32 Intel Xeon Skylake cores running at 2.1 GHz, 256 GB RAM, and one NVIDIA Tesla T4 GPU.

The results of the classification on the test sets are reported on Table 16. The results for *WH*+*TW* are averaged on the ten samples. We also compute the percentage gain of CNNs, RNNs and BERT w.r.t. the best bag-of-words classifier for each dataset. From this results, it emerges that the datasets that take the greatest advantage on the usage of recurrent neural networks and language models are *SENS2*, *SENS3* and *OMC* (the gain is between 10.26% and 14.71%), while the maximum improvement for *WH*+*TW* is 8.80%. It is worth noting that the performance of BERT on *OMC* are similar to those achieved by using the dictionary-based features (see Sect. 4.2) and significantly lower than those achieved by the same model on *SENS2* and *SENS3*. One possible explanation for this phenomenon is that the posts in this dataset deal with a limited number of very specific topics by construction. We recall, in fact, that its posts have been extracted from some targeted subreddits mentioning few very specific terms (see Sect. 3.2). As a consequence, a language representation model like BERT does not help improve classification results to a great extent. *SENS3*, instead, also has the highest F1-score using BERT (0.89), but it is worth recalling that this dataset has less than half the posts of all other datasets. Instead, the high performances achieved by BERT on *WH*+*TW* can be also explained by the fact that sensitive and non-sensitive posts are derived from two different microblogging platforms. Although this point is out of the scope of our work, the choice of a particular social media platform (especially when it promotes anonymous contents) may have an impact on both the lexicon and the language style adopted by the users. Finally, CNNs are less effective than RNNs and BERT. In *WH*+*TW*, they perform similarly as or even worse than any bag-of-words models. More detailed classification results for BERT are given in Table 17.

**Table 16:** Classification results (macro-averaged F1-scores and percentage gain w.r.t the best bag-of-word classifier)

Dataset	Classifier	F1-score	Gain
SENS2	BoW-LR	0.68	–
	BoW-RF	0.67	–
	BoW-SVM	0.68	–
	CNN1	0.73	7.35%
	CNN2	0.73	7.35%
	CNN3	0.72	5.88%
	CNN4	0.71	4.41%
	RNN1	0.77	13.24%
	RNN2	0.77	13.24%
	BERT	0.78	**14.71%**
SENS3	BoW-LR	0.73	–
	BoW-RF	0.73	–
	BoW-SVM	0.78	–
	CNN1	0.81	3.85%
	CNN2	0.81	3.85%
	CNN3	0.60	−11.76%
	CNN4	0.81	3.85%
	RNN3	0.87	11.54%
	RNN4	0.86	10.26%
	BERT	**0.89**	14.10%
OMC	BoW-LR	0.60	–
	BoW-RF	0.59	–
	BoW-SVM	0.60	–
	CNN1	0.63	5.28%
	CNN2	0.64	5.95%
	CNN3	0.65	8.29%
	CNN4	0.65	8.07%
	RNN1	0.65	11.67%
	RNN2	0.65	11.67%
	RNN3	0.66	13.33%
	RNN4	0.67	14.39%
	BERT	0.68	13.32%
WH+TW	BoW-LR	0.80 ± 0.01	–
	BoW-RF	0.78 ± 0.01	–
	BoW-SVM	0.81 ± 0.01	–
	CNN1	0.77 ± 0.03	−3.62 ± 3.89
	CNN2	0.78 ± 0.03	−4.51 ± 4.41
	CNN3	0.75 ± 0.07	−7.32 ± 8.31
	CNN4	0.68 ± 0.15	−15.15 ± 18.92
	RNN1	0.83 ± 0.02	2.59 ± 1.78%
	RNN2	0.83 ± 0.01	3.08 ± 1.48%
	RNN3	0.83 ± 0.01	3.28 ± 2.00%
	RNN4	0.84 ± 0.02	3.85 ± 1.85%
	BERT	0.88 ± 0.01	8.80 ± 1.55%

**Table 17:** Detailed classification results (F1-score) using BERT. Results on WH+TW are averaged on ten samples

Dataset	F1(sens.)	F1(non-sens.)	F1(macro)
SENS2	0.73	0.83	0.78
SENS3	0.85	0.92	0.89
OMC	0.75	0.61	0.68
WH+TW	0.85 ± 0.01	0.91 ± 0.01	0.88 ± 0.01

To measure the generalization strength of the classification models, we conduct the following additional experiment. We train the classification models on the training set of each dataset, but instead of testing them on the respective test set, we use every other entire dataset as test set. Hence, for instance, every model learnt on the training set of *SENS2* is tested on the entire *SENS3*, *OMC* and *WH*+*TW* datasets and viceversa. To prevent any bias, when using *SENS3* (resp. *SENS2*) as test set, instances that are also present in the training set of *SENS2* (resp. *SENS3*) are removed. In Table 18 we report the macro-averaged F1-scores computed on the test sets reported in the columns using the training sets reported in each row. We only show the results for SVM trained on the bag-of-word representation and BERT. Interestingly, when BERT is trained on *SENS2*, its performances are good when tested on *SENS3* too. Nonetheless, this is not that surprising, because *SENS3* is a subset of *SENS2* with less uncertainty on the class labels provided by the annotators (we recall that, in *SENS3*, the annotators’ agreement is maximum). However, the most interesting results are the ones obtained by the classifier trained on *SENS2* and tested on *WH*+*TW*, and viceversa. In this cases, the training and test sets are from completely different sources, and BERT trained on *WH*+*TW* has even worse performances than the bag-of-words model when tested on *SENS2*. Instead, BERT trained on *SENS2* achieves noticeably higher performances. It is worth noting that the difference in performances is the highest among all pairs of diverse datasets: in fact, the F1-score is 13% higher for BERT trained on *SENS2* and tested on *WH*+*TW* than for the opposite configuration. The performances of *OMC* on *WH*+*TW* with BERT are sensibly worse than those achieved by *SENS2*, although its performances on *SENS2* and *SENS3* are higher than those obtained by our datasets on the entire *OMC* dataset. This could be the consequence of the better representation provided by the training set of *OMC*, in particular for the sensitive class. In fact, the value of the F1-score for the sensitive class is 0.56 when the instances of *SENS2* are predicted with BERT trained on the training set of *OMC*, while, for the opposite configuration, the F1-score is 0.39. For the pair of datasets composed by *SENS3* and *OMC*, the same scores are, respectively, 0.57 and 0.36. It is worth noting that BERT trained on *WH*+*TW* achieves sensibly higher performances when tested on *OMC* rather than on *SENS2* or *SENS3*. This confirms that the type of sensitivity captured by *OMC* and *WH*+*TW* are similar. For further analysis, we also conduct the same experiment with dictionary-based features (see Sect. 4.2), using the Random Forest classifier (*DICT-RF* in Table 18). The results show that the models trained on *OMC* and *WH*+*TW* do not perform well on our datasets (the F1-score are between 0.19 and 0.33). Instead, the same models achieve better performances on their reciprocal test sets (macro-averaged F1-scores are 0.52 and 0.54), confirming that those datasets address similar problems (i.e., a more specific concept of self-disclosure than ours).

**Table 18:** Cross-classification results (macro-averaged F1-scores). Classifiers are trained on the datasets reported in the row, and tested on the datasets reported in the columns

Dataset	Class.	SENS2	SENS3	OMC	WH+TW
SENS2	DICT-RF	–	0.66	0.38	0.46 ± 0.00
	BoW	–	0.75	0.44	0.50 ± 0.00
	BERT	–	0.90	0.50	0.58 ± 0.00
SENS3	DICT-RF	0.63	–	0.37	0.44 ± 0.00
	BoW	0.64	–	0.42	0.47 ± 0.00
	BERT	0.74	–	0.48	0.51 ± 0.00
OMC	DICT-RF	0.33	0.21	–	0.52 ± 0.01
	BoW	0.51	0.51	–	0.55 ± 0.00
	BERT	0.56	0.59	–	0.52 ± 0.01
WH+TW	DICT-RF	0.29 ± 0.00	0.18 ± 0.00	0.54 ± 0.01	–
	BoW	0.48 ± 0.01	0.47 ± 0.02	0.50 ± 0.01	–
	BERT	0.45 ± 0.01	0.44 ± 0.01	0.57 ± 0.01	–

## Discussion of the results

In this section, we discuss more in detail the results of the experiments described in Sects. 4 and 5 and draw some generalized conclusions.

In our paper, we have performed many different data analysis tasks with the aim of investigating whether state-of-the-art approaches to self-disclosure detection in texts and the related text corpora, which have made available to the public, are adapted to identify privacy-sensitive posts shared in general purpose social media. Our main target is the typical social media post, which, in principle, may deal with arbitrary topics, and is communicated to different kinds of audiences, both in terms of extension (the number of profiles that can read the post) and type (close friends, acquaintances, general public). So far, the problem has been addressed by assuming that sensitive posts are published anonymously [15–17], or by considering a less general problem called self-disclosure [11]. In the experiments, not only have we shown the limitations of both approaches, but we have also pointed out the drawbacks of existing text corpora that might be used to train classification models capable of determining whether a given text is sensitive or not. Such corpora, in fact, are extracted from microblogging or forum platforms under very specific sections (e.g., dealing with family life or intimate relationships). As a result, they are not able to capture sensitive contents with wider topic coverage. Furthermore, we have created a new text corpus, consisting of around ten thousand Facebook posts, each annotated by three experts. In our corpus, sensitivity has a broader definition than self-disclosure and we think that this better captures the actual privacy-sensitive content that can be found in general-purpose social media. More than that, we do not make any anonymity assumption, in line with recent studies on the privacy paradox [12] and privacy fatigue [13] that show that many users tend to underestimate or simply overlook their privacy risk when posting on social media platforms.

All our experiments confirm that tackling the problem of content sensitivity by leveraging anonymity solves a less general problem than ours. By addressing sensitivity directly, we show that dictionary-based or bag-of-words based approaches are not that effective. Sequential models as Recurrent Neural Networks and language models, instead, lead to more accurate analysis and predictions and, more interestingly, introduce a significant performance gain on text annotated according to criteria that are not mediated by the lens of anonymity. Interestingly, *OMC*, a dataset that is specifically annotated according to self-disclosure [11], does not take advantage of RNNs or BERT to such a great extent: the results of these deep learning algorithms are comparable with those obtained by Random Forests trained on lexical features. The general mild performances of all types of classifier on this dataset could be explained by the overrepresentation of the sensitive class (corresponding to posts containing some form of self-disclosure). Unfortunately, this is by design, also because the dataset has been published with a different objective (i.e., the study of affect in response to interactive content). More interestingly, the posts extracted according to the anonymity criterion (*WH*+*TW*) and those extracted following the classic definition of self-disclosure (*OMC*) share some common properties, as testified by the cross-classification results (Table 18) and the mild correlation of the relevant feature for the logistic regression classifier (Table 13). This is probably the result of the particular choice of sources for the posts composing the sensitive class of those corpora (a subreddit on family relationships for *OMC* and Whisper for *WH*+*TW*). Finally, our experiments have shown that, for our datasets, only RNNs and BERT provide a significant performance boost. This phenomenon can be explained by the fact that, in general purpose social media, the context of a word/sentence (well captured by transformer-based language models) is more adapted to model the sensitivity of a post than simple lexical features. It is worth noting that BERT achieves good performances on *WH*+*TW* too. However, in this case, its performances could be biased by the fact that sensitive and non-sensitive posts are extracted from two different social media and, consequently, the network is not learning how to detect the sensitivity of a post, but, rather, the source of it. Although deserving further investigations, we leave this point for future research work.

Despite the results obtained and their analysis largely confirm our hypotheses, the extent of our work is in part limited by the fact that we have not controlled data acquisition, but, instead, rely on a corpus of Facebook posts collected ten years ago for different research purposes (i.e., predicting some psychological traits of users according to their behavior on the well known social network). Currently, it is not possible to collect such data, as Facebook has been limiting the amount of information that can be obtained by using its API since 2015. Nevertheless, it is the only available dataset composed of the so-called profile status updates. Other available Facebook posts are crawled from public pages, so they could hardly fit our objectives. Moreover, although we think that our work could foster further research on related topics, its impact is mitigated by the rapid changes in the social media world. Currently, the most popular social platforms (e.g., Instagram, TikTok) are designed for sharing multimedia content such as pictures and short videos. Although many results on text content presented in this paper (and in other similar research works) can be adapted or transferred to multimedia posts, new efforts should be undertaken to detect sensitive contents in pictures and videos accurately.

## Conclusion

With the final goal of supporting privacy awareness and risk assessment, we have introduced a new way to address the problem of sensitivity analysis of user-generated content without explicitly considering the so-called anonymity assumption. We have shown that the “lens of anonymity” could indeed distort the actual sensitivity of text posts. Consequently, differently from state-of-the-art approaches, we have measured the sensitivity directly, and we have collected reliable sensitivity annotations for an existing corpus of around ten thousand social media posts. In our experiments, we have shown that our problem is more challenging than anonymity-driven ones, as lexical features are not sufficient for discriminating between sensitive and non-sensitive contents. Moreover, we have also investigated how the problem of self-disclosure is related to content sensitivity analysis, and show that existing text corpora are not adequate to analyze the sensitivity of posts shared in general purpose social media platforms. Instead, recent sequential deep neural network models may help achieve good accuracy results. Our work could represent a new gold standard in content sensitivity analysis and could be used, for instance, in privacy risk assessment procedures involving user-generated content.^6^

On the other hand, our analysis has also pointed out that predicting content sensitivity by simply classifying text can not capture the manifold of privacy sensitivity with high accuracy. So, more complex and heterogenous models should be considered. Probably, an accurate sensitivity content analysis tool should consider lexical, semantic as well as grammatical features. Topics are certainly important, but sentence construction and lexical choices are also fundamental. Therefore, reliable solutions would consist of a combination of computational linguistic techniques, machine learning algorithms and semantic analysis. Finally, the success of picture and video sharing platforms (such as Instagram and TikTok), implies that any successful sensitivity content analysis tool should be able to cope with audiovisual contents and, in general, with multimodal/multimedia objects (an open problem in sentiment analysis as well [49]).

## Data Availability

The datasets used and analysed during the current study are available from the corresponding author on reasonable request.

## Footnotes

1 https://github.com/kj2013/claff-offmychest

2 http://whisper.sh/

3 https://github.com/Mainack/whisper-2014-2016-data-HT-2020

4 Available at https://nlp.stanford.edu/projects/glove/

5 Available at https://keras.io/ and https://scikit-learn.org/

6 The source code used in our experiments is available online at https://github.com/bioglio/sens_prediction.
